# Investigating factors that influence the practice of exclusive breastfeeding among mothers in an urban general hospital in Ghana: a cross-sectional study

**DOI:** 10.1186/s12905-023-02164-y

**Published:** 2023-01-18

**Authors:** Baaba Dadzie, Fidelis Bayor, Abdul-Razak Doat, Jamilatu B. Kappiah, Collins Adombire Akayuure, Aubrey A. Lamptey, Vida Nyagre Yakong, Sylvanus Kampo

**Affiliations:** 1 School of Medicine, University of Health and Allied Sciences, Ho, Ghana; 2 Department of Physiology, School of Medicine and Dentistry, University of Ghana, Accra, Ghana; 3 Department of Nursing, School of Nursing and Midwifery, C. K. Tedam University of Technology and Applied Sciences, Navrongo, Ghana; 4 Department of Pediatric Critical Care, St. Jude Children’s Research Hospital, 262 Danny Thomas Place, Memphis TN, Accra, Ghana; 5 Department of Preventive Health Nursing, School of Nursing and Midwifery, University for Development Studies, Tamale, Ghana; 6 Department of Anaesthesia and Intensive Care, School of Medicine and Dentistry, C. K. Tedam University of Technology and Applied Sciences, Navrongo, Ghana

**Keywords:** Exclusive breastfeeding, Six months, Children, Mothers, Attitude, Level of knowledge

## Abstract

**Background:**

In Ghana, only 52% of mothers exclusively breastfeed their babies and the rate of increase has been steadily slow across all geographical areas of Ghana. The purpose of this study was to determine the various factors that influence exclusive breastfeeding (EBF) among mothers who visited the child welfare clinic at the Tema General Hospital, Accra, Ghana.

**Methodology:**

This descriptive cross-sectional study was carried out at the Child Welfare Clinic of the Tema General Hospital, Accra, Ghana. A random sampling technique was used to recruit mothers with children between the ages of 6 months and 24 months attending the Child Welfare Clinic. Mothers were interviewed with the aid of a structured questionnaire.

**Results:**

Out of the 222 of mothers interviewed, 68.8% of them exclusively breastfed their infants up to 6 months. Mothers who have good knowledge were more than 3 times (AOR = 3.484, 95% CI 1.200, 10.122, *P* = 0.022) likely to breastfeed their children exclusively. Those who had positive attitudes towards EBF were about 4 times (COR: 4.018, 95% = 1.444, 11.181, *P* = 0.008) more likely to exclusively breastfeed than those who had poor attitudes towards EBF. Also, mothers whose spouses complained about EBF were about 3 times (AOR: 2.655, 95% CI 0.620, 11.365, *P* = 0.018) at increased odds of not exclusively breastfeeding their babies.

**Conclusions:**

High rate of EBF among mothers who visited the child welfare clinic was found. The mothers' level of knowledge and attitude towards EBF significantly influenced the 6 months of EBF. Spouses also showed a high influence on whether or not mothers should exclusively breastfeed their babies.

## Introduction

The World Health Organization recommends that mothers exclusively breastfeed their babies for the first 6 months after birth [1]. EBF is defined as providing infants with only breast milk as their sole source of nutrition, with the exception of necessary oral rehydration solution, drops, and syrups of vitamins, minerals or medications [2, 3]. The benefits associated with EBF include: healthier eating habits, a shorter hospital stay, more favourable weight gain, a lower body mass index, less adiposity, lower total cholesterol values, better cognitive and behavioural development, and metabolic level stability in children with metabolic disorders [4]. EBF plays a significant role in reducing infant morbidity and mortality as well as certain illnesses such as type II diabetes [4–7]. The American Academy of Paediatrics considers breastfeeding as the ideal and optimal source of nutrition in the first year of life, most importantly the first half, and recommends that mothers and caregivers exclusively breastfeed for the first 6 months after a baby is born, followed by complementary feeding with solid foods [8].

According to the United Nations International Children’s Emergency Fund (UNICEF), the infant mortality rate in Ghana as of 2019 stood at 34 deaths per 1000 live births and the under 5 mortality rate stood at 46 deaths per 1000 live births [9]. In 2008, infections and malnutrition accounted for a huge proportion of the causes of infant mortality of which a reasonable number could have been prevented with EBF [10]. The Ghana Demographic and Health Survey conducted in 2008 showed that 63% of infants below 6 months were being exclusively breastfed [10]. The survey re-conducted in 2014 however showed a decrease in this number, as only 52% of infants below 6 months were exclusively breastfed [11]. This represents an 11% decrease in the EBF rate from 2008 to 2014.

In order to achieve Sustainable Development Goals 3.2 which tackles preventable deaths in neonates and children under five, efforts need to put in place globally, and even more specifically in developing countries like Ghana, in the area of EBF as it contributes enormously to reducing mortality in infants. Knowing the wide array of benefits EBF provides, it is of relevance to decipher ways and means to achieve maximum numbers in terms of EBF among mothers. This will help reduce drastically the infant mortality rates, infectious illnesses that could have been otherwise prevented by EBF as well as malnutrition in their children.

Despite extensive research and the advantages associated with EBF, the practice remains low globally, with rates declining fastest in developing nations due to the significant influence by contextual and environmental factors [4, 5]. Evidence shows a significant difference in EBF between urban and rural areas. For example, Astika et al. [12] discovered that mothers who reside in urban areas were more likely to commence early breastfeeding and breastfeed exclusively than those in rural areas. Also, low patronage were attributed largely to sociodemographic and cultural practices in Ghana and other Sub-Saharan African countries, as well as low levels of knowledge, misconceptions, maternal age and health conditions, antenatal care services, the economic situation of the family, access to information, and employment issues [13–18]. In order to maximize positive perception and attitude toward the practice of EBF in these areas, educational interventions to help women better understand the benefits of EBF is required. While studies on exclusive breastfeeding in Ghana have focused on the knowledge level, socioeconomic and cultural practices as being the major factors affecting the practice of EBF [13, 16–19], information on the effect of family support from parents, spouse and healthcare professionals on the practice of EBF has remained inadequate.

According to the theory of planned behaviour, the intention to carry out a specific behaviour is said to be the primary motivator behind an individual's particular behaviour. These intentions are presumptively intended to capture the driving forces behind motivation, including attitude, subjective norm (perceived expectations from others and how they value them), and perceived behaviour control (individual's level of knowledge or competences) [20]. This has the power to affect how decisions are made. As a result, motivational factors like a maternal knowledge, attitude, and perceived expectations from others, such as family support, may affect her intentions or willingness to exclusively breastfeed her infants. This study intends to provide additional information by assessing the factors including level of support from family members influencing the practice of EBF among mothers in an urban general hospital in Ghana. This crucial information has not been captured in the studies conducted so far in Ghana creating a knowledge gap that this study seeks to fill. It will also contribute to scientific knowledge and enable formulation of relevant, attainable and realistic policies aimed at increasing the rates of EBF.

## Methods

### Research design

A retrospective quantitative cross-sectional study design using researcher administered questionnaire was employed to describe the factors that influence EBF among mothers visiting the Tema General Hospital, Ghana. This study adopted the theory of planned behaviour [20] as its conceptual framework. This allowed access to information on the practice of exclusive breastfeeding among the study population, knowledge, level of support provided as well as the attitude of these mothers toward EBF.

### Study area

The study was conducted at the Child Welfare Clinic of the Tema General Hospital, a highly patronized district hospital located in the Greater Accra Region of Ghana. This healthcare facility is equipped with the necessary infrastructure to deliver both primary and specialist health care to all clients within the region and its environs. The Child Welfare Clinic undertakes activities such as growth monitoring of children, vaccinations and other services including birth registry. Most children visit this facility from birth to 5 years when vaccinations are completed.

### Inclusion and exclusion criteria

The inclusion criteria were made up of mothers who visited the Child Welfare Clinic of the Tema General Hospital. All registered postnatal mothers and parent/legal guardian with children aged between 6 and 24 months who were of sound mind and competent enough to give assent/consent were included in the study. This was done to obtain information from those who had completed the recommended 6 months of EBF and was limited to mothers with babies under 24 months in order to reduce recall errors and biases. We excluded all mothers with conditions which do not support breastfeeding such as babies with an established diagnosis of galactosemia, babies of deceased mothers, mothers who had mastectomy, those actively receiving cancer treatment, taking drugs (e.g. amphetamines, statins and antidepressants), those with active TB and mothers with Human T-lymphotropic virus as well as all eligible mothers who declined to participate in this study. Mothers with babies less than 6 months were exempted because EBF is recommended for the first 6 months and it cannot be ascertained as to whether they would practice EBF for the period until their babies turned 6 months.

### Sampling procedure

Using a simple random sampling technique, all eligible mothers who had their written informed consent taken were required to randomly pick confidentially prepared slips that had either a YES or NO inscribed. Only qualified participants who met the selection criteria and picked a slip with YES inscribed were enrolled for the study.

The sample size for the study was estimated using the Cochran formula below;

$${\text{N}} = \left( {\text{Z}} \right)^{2} {\kern 1pt} *{\kern 1pt} {\text{P}}\left( {1 - {\text{P}}} \right)/{\text{D}}^{2}$$

where N = sample size to be determined, Z = Z score (reliability coefficient) of 1.96 at 95% confidence level, P = the estimated proportion of the population who practiced exclusive breastfeeding. This was determined using a single population proportion from a study by Boakye-Yiadom et al., 2016 [21] to be 84.3% = 0.85, and D = margin of error of 5% = 0.05. The sample size calculated was 202. Assuming a non-response rate of 10%, the total sample size required for the study was 222.

### Data collection process

The total time used for this study was 9 weeks, starting from 21st of June to 20th of August, 2021. The first 5 weeks were used for data collection and the subsequent four for analysis. After obtaining ethical approval from the Research Ethics Committee of the University of Health and Allied Sciences (UHAS-RECA.12 [171] 21-21), permission to commence data collection was sought from the clinical coordinator and the in-charge of the Child Welfare Clinic of the Tema General Hospital, Ghana. Written informed consent was also obtained from the individual mothers who met the selection criteria after providing them with adequate explanations regarding the aims of the study. For participants who were below 18 years, informed consent was sought from their parents/legal guardians who accompanied them.

Following informed consent and recruitment, study participants were interviewed using a standard structured self-administered questionnaire which was developed and validated by the authors for this study. The validity of the questionnaire was determined by the adoption of the checklist/guideline by the Centers for Disease Control and Prevention (CDCP), 2014 [22], Global Opinion Panels [23] and the breastfeeding self-efficacy scale [24] and was pre-tested on five participants which served as a pilot study and the reliability determined through a review with two public health physicians and a paediatrician. The study participants were approached and assessed for their eligibility to participate in the study following the determination of the age of their children between 6 to 24 months. In all, a total of 222 eligible mothers were enrolled in the study. The questionnaire was prepared in English and had it translated and explained to mothers in their local language where necessary. Questionnaires were numbered and coded prior to data collection. The questionnaires sought to provide information on the various factors that influence EBF among mothers. This was done by assessing the practice of EBF among the study population, knowledge of EBF, the attitude of mothers toward EBF as well as the level of support received from spouses.

### Measurement of variables

#### Independent variables

Age of mother and baby, sex of the baby as well as the level of education of the mother, marital status of the mother, religion, occupation, place of residence and the number of children she had were defined as independent variables. The occupation of the mothers was then defined as formal, informal or unemployed with those being unemployed including students. The place of residence was also categorised into various districts. Also, questions were asked on what hospital the mothers attended for antenatal care, where they delivered and the gestational age at which they delivered. The hospitals mothers attended for antenatal care were categorised into either a government facility or a private facility. For the birth weight, all babies born with birth weight from 2.5 to 4.5 kg were noted to have normal weight whereas all those less than 2.5 kg or more than 4.5 kg were considered to have a low birth weight and a high birth weight respectively. The current weight of the children was assessed using the weight for age chart for the various sexes and all those between the -2 standard deviation and + 2 standard deviation were considered to have normal weight. Those above + 2 standard deviation and those below -2 standard deviation were evaluated to be overweight and underweight respectively.

### Dependent/outcome variables

The WHO criteria or indicators for assessing infant feeding practices, Geneva, 2021, were used to assess EBF [25]. The main dependent variable was mothers’ practice of EBF. EBF was defined as the babies receiving only breast milk as a source of nutrition. The first type of feed was also asked together with whether they fed the first yellowish breast milk or not and if they fed their babies with other foods or fluids. Mothers were also asked when they stopped breastfeeding and if they were still breastfeeding at the time of the study.

To measure mothers’ level of knowledge on EBF, they were asked about whether or not they had heard of EBF and subsequently about where they heard about it from and also when they heard about it. The respondents were asked when one should initiate breastfeeding after delivery and when one should start giving water and then food to their children. They were also asked if breast milk alone was enough for the babies in the first 6 months and what one should do with the first breast milk. Knowledge about the benefits of EBF was assessed. The overall level of knowledge of mothers was considered to either be good or poor. The criteria for assessing knowledge on EBF was used based on literature [23, 25–28].

A score from zero to four implied you had poor knowledge of EBF whereas a score of more than four implied a good knowledge of exclusive breastfeeding. The level of support obtained from the family, community and health professionals was ascertained. This was done by finding out whom they stayed with, then who helps take care of the baby followed by who provides money to take care of the baby. Further questions were asked about whether the spouse or the parents of the respondents complained about EBF.

Finally, attitude of the mothers towards exclusive breastfeeding was determined. The questions required answers using the 3-point Likert scale. The questions began with asking the mothers if they found it difficult exclusively breastfeeding for 6 months and continued by asking if they felt it was okay to give their children formula if they were not producing enough breast milk. They were also asked if it was okay to give complementary feeds before 6 months if the baby was not satisfied after feeds or if it was okay to give water to their babies before 6 months if the babies were thirsty. Again, they were asked if they found it difficult to breastfeed in public and if they felt confident expressing breast milk to be given to their children in their absence. To evaluate mothers as having a good or poor attitude towards EBF, choosing disagree scored one point while disagree or unsure scored zero point. Cumulatively, mothers could score from zero to seven. A mother with a score less than four was deemed to have a poor attitude towards EBF and one who scored four through to seven was noted to have a good attitude towards EBF.

### Data analysis

Data were double entered into Microsoft excel, validated for entry errors and exported onto Statistical Package for Social Sciences Software (SPSS) version 20.01 (IBM Corporation, Armonk, NY, USA) for statistical analysis [29, 30]. The results were presented as means, frequencies and tables. The confidence interval was 95% and considered statistically significant at *P* < 0.05. The frequency distribution was done for all variables. The Pearson chi-square test was used to test the significance of the association between the practice of EBF and knowledge, attitude, support received and complaints of relatives. Factors found to have significant associations were analyzed using a multiple logistic regression model which was used to calculate the odds ratio and confidence interval. *P-values* of variables in the chi-square table with only significant terms as predictors of EBF was the criteria for variable selection to fit the multiple logistic regression model. Socioeconomic indicators such as income level, maternal health condition or mental state relation, parity, maternal age, gestational age, educational level, occupation and employment issues were perceived as major confounders to the multiple logistic regression model. These were chosen because many previous studies [14, 17–20] have reported the influence of socio-economic and cultural factors on the practice of EBF. Missing data was managed using a listwise or case deletion. To address data bias, multiple people were used to code the data; results were reviewed and were verified from other data sources and compared with other studies’ results.

## Results

### Baseline information of participants

A total of 222 mothers with their children were recruited for this study of which 30 (13.5%) were within the age range of 16–25 years, 134 (60.4%) were within the age bracket of 26–35 years, and 58 (26.1%) aged between 36 and 45 years (Table 1). The mean age was 32 years. The average age of the children of mothers recruited was 11 months with 53 in every 100 of them being females. The average weight of the children at birth was 3.2 kg. With regards to the educational background of the mothers, 53 (23.9%) of them had Junior high school education, 82 (36.9%) had senior high school education, 65 (29.3%) had Tertiary education, whereas 14 (6.3%) had primary education (Table 1). The parity of 205 (92.3%) of the mothers was between 1–4, and 17 (7.7%) of them were greater than 4 (Table 1). 187 (84.2%) of the mothers were employed, while 35 (15.8%) of them were unemployed (Table 1). Those who were residents of Ashaiman constituted the majority, 104 (46.9%) of our study participants, 98 (44.1%) were residents at Tema and 20 (9%) were residents at other places in Accra. Also, 204 (91.4%) of the participants said they had term deliveries compared to the 19 (8.6%) preterm deliveries reported (Table 1).

**Table 1:** Baseline information of participants

Variables	Frequency	Percentage (%)
*Age of mother*		
16–25	30	13.5
26–35	134	60.4
36–45	58	26.1
*Level of education*		
None	8	3.6
Primary	14	6.3
JHS	53	23.9
SHS	82	36.9
Tertiary	65	29.3
*Marital status*		
Not Married	49	22.1
Married	173	77.9
*Religion*		
Christian	202	91
Muslim	20	9
*Occupation*		
Unemployed	35	15.8
Employed	187	84.2
*Parity*		
1–4	205	92.3
> 4	17	7.7
*Place of delivery*		
Hospital	219	98.7
Home	3	1.3
*Residence*		
Tema	98	44.1
Ashaiman	104	46.9
Others	20	9
*Gestational age*		
Term	203	91.4
Pre-term	19	8.6

The average age of the children of mothers recruited was 11 months with 53 in every 100 of them being females. The average weight of the children at birth was 3.2 kg

### Knowledge on exclusive breastfeeding

A vast majority (94.1%) of mothers were knowledgeable of EBF. About 218 (87.9%) reported the hospital was their primary source of information about EBF. Compared to the 50 (22.5%) mothers who thought breast milk during the first 6 months does not give appropriate nourishment to their babies, more than three-quarters (76.1%) of the mothers believed that EBF was a source of adequate nutrition to their babies. Out of the mothers who felt they were not producing enough breast milk for their babies, 46 (20.7%) continued breastfeeding, 56 (25.2%) reported to the hospital, and 20 (9.1%) provided supplemental feedings (Table 2).

**Table 2:** Knowledge on exclusive breastfeeding

Variables	Frequency	Percentage (%)
*Source of information		
Hospital	218	87.9
Television	8	3.2
Internet	9	3.6
Family	5	2.1
Friends	8	3.2
*Adequacy of breastmilk for first 6 months*		
Adequate	169	76.1
Inadequate	50	22.5
Missing	3	1.4
*Actions taken by mothers who perceived not to have enough breastmilk*		
Continue breastfeeding	46	20.7
Give supplementary feeds	20	9.1
Report to hospital	56	25.2
Others	96	43.2
Missing	4	1.8
*Level of knowledge*		
Good	209	94.14
Poor	13	5.86

A vast majority (94.1%) of mothers were knowledgeable of EBF

^*^Multiple choice question

### Practice of exclusive breastfeeding

To investigate the practice of EBF among mothers, we first determined mothers who practised EBF for the recommended period. The data showed that 146 (65.8%) mothers practised EBF for the recommended period compared to the 76 mothers (34.2%) who did not. 219 (98.6%) of the mothers fed their babies with colostrum. The study indicated that 115 (51.8%) of mothers initiated breastfeeding immediately after birth, 40 (18.0%) initiated breastfeeding the day after birth, and 66 (29.7%) of the mothers-initiated breastfeeding 2 or more days delivery (Table 3). The initial food given to babies immediately after birth by mothers were; breast milk (85.5%), formula (14.0%) and only one mother (0.5%) gave glucose water.

**Table 3:** Practice of exclusive breastfeeding

Variables	Frequency	Percentage (%)
*The time when initiated breastfeeding*		
Not at all	1	0.5
Immediately after birth	115	51.8
Immediately after a day of birth	40	18.0
After days of birth	66	29.7
*Practised exclusively breastfeeding for 6 months*		
Yes	146	65.8
No	76	34.2
*Initial food given to babies immediately after birth*		
Breast milk	190	85.5
Formula	31	14.0
Glucose water	1	0.5
*Colostrum*		
Yes	219	98.6
No	3	1.4

The data showed that 146 (65.8%) mothers practised EBF for the recommended period compared to the 76 mothers (34.2%) who did not

### Level of support

In our study, 196 (88.3%) lived with their spouses. Up to 79.7% of the respondents had support from their spouse, parents, and extended family members when they had difficulties with EBF and 95.5% of the mothers had their income provided by their spouses (Table 4). According to the study’s findings, 55 (24.8%) of those who complained about EBF were their parents and 22 (9.9%) were their spouses.

**Table 4:** Level of support

Variables	Frequency	Percentage (%)
*Whom they lived with*		
Alone	9	4.05
Spouse	196	88.29
Parents	23	10.36
Extended family member	10	4.5
*Who helped take care of the infant*		
No one	46	20.72
Spouse	92	41.44
Parents	64	28.83
Extended family member	21	9.46
Others	15	6.76
*Who provided the income?*		
Self	56	22.23
Spouse	212	95.5
Parents	8	3.6
*Who complained about the practice of exclusive breastfeeding?*		
Parents	55	24.77
Spouse	22	9.91

Up to 79.7% of the respondents had support from their spouse, parents, and extended family members when they had difficulties with EBF and 95.5% of the mothers had their income provided by their spouses

### Attitude towards exclusive breastfeeding

**Fig. 1:**
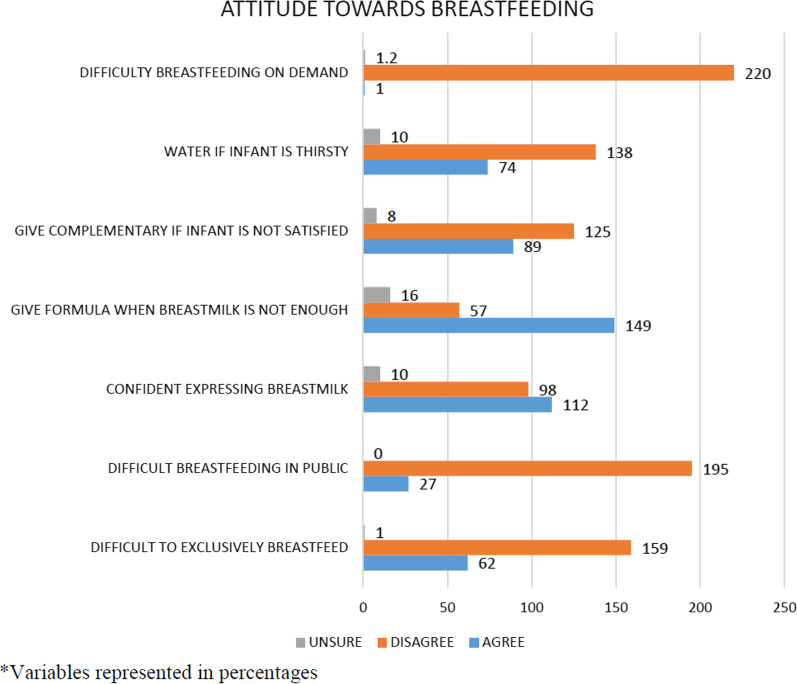
Attitude towards exclusive breastfeeding. Mothers had good attitude towards EBF. However, one out of every five mothers had a poor attitude towards EBF

Among the respondents interviewed for this study, 67.1% (n = 149) agreed to give formula when they feel that the breast milk is not enough for their babies. 50.9% (n = 112) of the mother were confident expressing their breast milk and 40.1% (n = 89) agreed that they will give complementary feeding if they feel that their babies are not satisfied with the breast milk (Fig. 1).

### Association between maternal factors and the practice of EBF

To determine the various factors that influence the practice of EBF among mothers visiting the Child Welfare Clinic of the Tema General Hospital, a bivariate analysis was done using Chi-square (x^2^) test. The practice of EBF was significantly associated with 69.4% (n = 145) of the women who had adequate level of knowledge compared to those who did not adhere to EBF for 6 months (*p* < 0.001, x^2^ = 20.684). 75.1% (n = 127) of the mothers who practiced EBF said giving breast milk alone for 6 months is enough compared to those who did not practice EBF (*p* < 0.001, x^2^ = 29.018). Furthermore, while EBF was significantly associated with positive attitude toward EBF (*p* < 0.001, x^2^ = 51.917), only about 41.9% (n = 26) had a difficulty with EBF (*p* < 0.001, x^2^ = 24.212), 53.0% (n = 79) agreed that they will give their babies formula (*p* < 0.001, x^2^ = 32.862 and 44.9% (n = 40) were okay with complementary foods (*p* < 0.001, x^2^ = 28.691). Although there was no significant associations between EBF and support received (*p* = 0.881, × 2 = 0.022), 68.2% (n = 15) had their spouse complained (*p* < 0.001, x^2^ = 12.500) (Table 5).

**Table 5:** Bivariate analysis

Characteristic	Exclusive breastfeeding (EBF) for 6 months (N = 222)			
	Yes (%)	No (%)	Chi-Squared	*P*-Value
*Knowledge on EB*			20.684	< 0.001
Adequate	145 (69.4)	64 (30.6)		
Inadequate	1 (7.7)	12 (92.3)		
*Breast milk enough for 6 months*			29.018	< 0.001
Yes	127 (75.1)	42 (24.9)		
No	19 (35.8)	34 (64.2)		
*Continue breastfeeding*			1.711	**0.191**
Yes	34 (73.9)	12 (26.1)		
No	112 (63.6)	64 (36.4)		
*Report to hospital*			0.325	**0.569**
Yes	12 (60.0)	8 (40.0)		
No	134 (66.3)	68 (33.7)		
*Food supplements*			0.849	**0.357**
Yes	34 (60.7)	22 (39.3)		
No	112 (76.7)	54 (71.1)		
*Attitude towards EB*			51.917	< 0.001
Negative	15 (28.3)	38 (71.7)		
Neutral	32 (61.5)	20 (38.5)		
Positive	99 (84.6)	18 (15.4)		
*Difficulty exclusive breastfeeding*				
Agree	26 (41.9)	36 (58.1)	24.212	< 0.001
Disagree	120 (75.5)	39 (24.5)		
*Okay formula*			32.862	< 0.001
Agree	79 (53.0)	70 (47.0)		
Disagree	53 (93.0)	4 (7.0)		
Unsure	14 (87.5)	2 (12.5)		
*Okay complementary foods*			28.691	< 0.001
Agree	40 (44.9)	49 (55.1)		
Disagree	100 (80.0)	25 (20.0)		
Unsure	6 (75.0)	2 (25.0)		
*Okay water*			0.272	0.602
Agree	29 (39.2)	45 (60.8)		
Disagree	114 (82.6)	24 (17.4)		
Unsure	3 (30.0)	7 (70.0)		
*Support received*			0.022	0.881
No	32 (33.7%)	63 (66.3%)		
Yes	44 (34.6%)	83 (65.4%)		
*Spouse complained*			12.500	< 0.001
No	61 (30.5)	139 (69.5)		
Yes	15 (68.2)	7 (31.8)		
*Parents complained*			2.871	0.090
No	52 (31.1)	115 (68.9)		
YES	24 (43.6)	31 (56.4)		

The practice of EBF was significantly associated with 69.4% (n = 145) of the women who had adequate level of knowledge compared to those who did not adhere to EBF for 6 months (*p* < 0.001, x^2^ = 20.684)

^*^p < 0.05 considered statistically significant using chi-square

### Multiple logistic regression analysis

The factors found to have significant associations were analysed using a multiple logistic regression. The model had an overall accuracy value of 89.6 with a *P*-value of 0.000. Multivariate analysis shows that mothers’ level of education was significantly associated with the practice of EBF. Mothers who have good knowledge were more than 3 times (AOR = 3.484, 95% CI 1.200, 10.122, *P* = 0.022) likely to exclusively breastfeed their children. Those who had positive attitudes towards EBF were about 4 times (COR: 4.018, 95% = 1.444, 11.181, *P* = 0.008) more likely to exclusively breastfeed as opposed to those who had poor attitudes towards exclusive breastfeeding. Also, mothers whose spouses complained about EBF were about 3 times (AOR: 2.655, 95% CI 0.620, 11.365, *P* = 0.018) increased odds not to exclusively breastfeed their babies (Table 6).

**Table 6:** Multiple logistic regression analysis

	Crude Odds Ratio		Adjusted Odds Ratio	
	OR (95% CI)	*P*-value	OR (95% CI)	*P*-value
*Knowledge of EB*				
Adequate	4.444 (1.25015.805)	0.021	3.484 (1.200, 10.122)	0.022
Inadequate	1		1	
*Attitude*				
Positive	4.018 (1.444, 11.181)	0.008	3.491 (0.892, 13.665)	0.073
Negative	1		1	
*Support received*				
No	1		1	
Yes	1.184 (0.490, 2.863)	0.708	1.147 (0.446, 2.952)	0.775
*Spouse complained*				
No	2.117 (0.649, 6.903)	0.021	2.655 (0.620, 11.365)	0.018
Yes	1		1	
*Parents complained*				
No	1.080 (0.403, 2.893)	0.878	0.940 (0.271, 3.256)	0.922
Yes	1		1	

Multivariate analysis shows that mothers’ level of education was significantly associated with the practice of EBF

1 = reference category

## Discussion

The purpose of this study was to determine the factors that influence exclusive breastfeeding among mothers who visited the Tema General Hospital. This was done to identify additional factors (e.g. mothers’ level of knowledge, practice of EBF and level of support from family and spouse) that influenced the practice of EBF among mothers who visited the child welfare clinic. In previous studies, significant determinants of exclusive breastfeeding (EBF) have been identified to include maternal attitude and knowledge, as well as socio-demographic and cultural factors [17, 18]. In order to reduce infant morbidity and mortality in settings with limited resources, mothers should be encouraged to breastfeed exclusively for the first 6 months [31, 32]. Also, EBF is well known to play a critical role in fostering infant development, immunity, and illness prevention [33, 34]. Key findings of this study were as follows:

Our study demonstrated that over 95% of the women had good knowledge of EBF before their last delivery. The mothers viewed that prevention of neonatal sepsis, provision of adequate nutrition, enhancement of brain development; family planning, reducing the financial burden, time saving and enhancing bonding between mother and baby were some of the benefits that could be derived from EBF. Almost 66% of the mothers practiced EBF for the first 6 months and of those who failed to adhere to EBF, 65% were due to insufficient breast milk. One-third of the mothers had complaints about EBF from either their spouse or parents although about 58% of support was from their spouse, parents, extended family members or health personnel when they had difficulties with EBF. Again, 76.13% of the mothers had a good attitude toward EBF.

It is important to note that good knowledge (perceived behavior control) about the benefits of EBF according to the theory of planned behaviour may influence positive attitude and the intention or desire to exclusively breastfeed [20]. This study demonstrated significant maternal knowledge and positive attitude of the mothers toward EBF. This good knowledge on EBF is reflected in their view that water and other complementary foods should be initiated after 6 months of EBF and that colostrum is ideal for initiating breastfeeding in infants. As several studies have shown, about 94.14% of the mothers had good knowledge on EBF among mothers vising the Tema General Hospital with a large percentage (98.64%) of them obtaining the information on EBF from hospitals [21, 35, 36]. The child welfare and antenatal clinics of the facility carry out educational talks to clients every morning on various topics including EBF and this could have played a role in the high level of knowledge these mothers had. Out of every five mothers interviewed, four thought breast milk alone was enough for the first 6 months which certainly plays a role in determining whether or not a mother introduces supplementary feeds before the 6 months is completed. Our findings also agree with Dukuzumuremyi et al. [1] in East Africa who reported that almost 96.2% of mothers knew about EBF with an awareness rate of 84.4% and 49.2% knew that EBF should be done for the first 6 months only. In their study, maternal attitude toward EBF was good [1]. In Zimbabwe, although the practice was low, Mundagowa et al. [37] found a significant good maternal knowledge and positive attitude toward breastfeeding. Both good maternal knowledge and positive attitude were also demonstrated by Hoseini et al. [38] in a cross-sectional study in Mashhad. In contrast, Haghighi and Varzandeh [39] in Iran found a low level of maternal knowledge although they had a good attitude toward breastfeeding. Ekambaram et al. [40] also found poor maternal knowledge, especially regarding the time of initiation of breastfeeding, colostrum feeding, and duration of EBF, expressed breast milk and continuation of breastfeeding while the baby is sick. However, significant correlations were found between higher maternal age, better maternal education, higher socioeconomic status and having received antenatal care from tertiary care centers and private practitioners. There is still a need for a programme, which support and encourage breastfeeding particularly at a primary care level, focusing more on younger, less well-educated women and those from lower socioeconomic class.

Again, the 76.0% positive attitude recorded in this study was significantly associated with adequate maternal knowledge, the attitude of the mother and whether or not the spouse complained about EBF. Also, mothers who have good knowledge had increased odds to exclusively breastfeed their children and those who had positive attitudes towards EBF were 4 times likely to exclusively breastfeed, while mothers whose spouses complained about EBF were 3 times likely not to adhere to EBF.

It is worth noting that having a good understanding of the benefits of breastfeeding has a bigger impact on EBF compliance. The mothers' high knowledge and positive attitude toward EBF, as in the current study, could be linked to their strong belief that exclusive breastfeeding is associated with significant benefits. Our findings were not different from Smith and Forrester [41] where EBF was said to minimize public health costs. Similarly, Allen and Hector [42] reported that EBF is associated with several health benefits similar to those reported by Couto et al. [4]. As a result, emphasizing the maternal benefits of EBF could encourage mothers to breastfeed their infants exclusively.

According to Oche et al. breastfeeding continues to be the most straightforward, healthiest, and affordable feeding method for almost all infants [43]. In their study, although the practice of EBF was found to be low (31%) in Sokoto state, Nigeria, the proportion of mothers who practiced EBF for the recommended 6 months was almost 66%, with one in every four women who start EBF stopping before the 6 months is up. This proportion is similar to the 64% recorded in 2013 across Ghana [44]. It was 14.2% higher than the estimated 52% by the Ghana Demographic and Health Survey in 2014 [11] but lower than the study by Boakye-Yiadom et al. in Mamprusi West by 18.5% which could be attributed to the mother-to-mother support group created in the area by non-governmental organizations. Those who failed to practice EBF, 65% were due to insufficient breast milk. The increased desire and willingness to comply with EBF practices among our study participants may be largely due to the good knowledge and understanding about the numerous advantages associated with EBF. Our finding on mothers’ practice of EBF was a little higher than those reported by Dukuzumuremyi et al.

The theory of planned behavior states according to Ajzen [20], intentions are what drive individual behaviors and are influenced by things like attitudes, subjective norms, and perceived behavioral controls. Therefore, depending on how accurately perceived behavior control represents actual behavior control; external factors such as level of support or expectations from family and healthcare professionals (subjective norm) may directly affect behavior regardless of the intention [20]. Our study also identified that mothers had about 58% supports from spouses, parents, close relations and healthcare professionals. Although few complaints from close relations were reported, support from immediate family members contributed enormously to building a strong mental and positive attitude, serving as a major determinant of the high patronage toward EBF among the mothers. On the other hand, studies in Ethiopia, Nigeria and Ghana reported that the major determinants of EBF were directly linked to maternal sociodemographic and economic status such as maternal age, maternal monthly earnings, level of education, employment status, mothers who delivered at a healthcare facility, antenatal attendance [34, 45, 46]. Even though 95.5% of the mothers who participated in this study were financially supported by their spouses, only two in every five of them were assisted by their spouse in taking care of the child. Two again out of the five mothers interviewed was assisted by other people besides their spouse implying that one out of every five of them did not receive any assistance in the care of the infants. However, there was no association between 6 months of EBF and mothers receiving support when they faced challenges EBF (*p* = 0.881). Again, there was no association between EBF and mothers whose parents complained about EBF (*p* = 0.090) unlike parts of the Greater Accra Region where grandmothers of the baby were the most powerful influencers [46]. However, spouses seemed to influence the practice of EBF the most in this study evidenced by a significant association between the practice of EBF and mothers whose spouses complained about EBF. Mothers whose spouses complained about EBF had 79.5% decreased odds of EBF. This could be due to poor knowledge on EBF of the spouses causing them to not appreciate the importance of this practice. Also, up to three out of five of the mothers did not receive help from their spouses when they had difficulties with EBF. This could mean that more than half of these spouses are not actively involved in the care of their babies and as such may not be able to make positive contributions towards the practice of EBF.

## Conclusion

In conclusion, a high rate of EBF was observed among mothers who visited the child welfare clinic at Tema General Hospital. The increased rate of practice and a positive attitude toward EBF was as a result of their significant knowledge of the advantages associated with EBF. Also, whether or not mothers should exclusively breastfeed their babies was highly influenced by support from their spouse, parents, and healthcare professionals. Therefore, midwives, nutritionists, and public health nurses should continuously educate mothers about EBF both before and after delivery at both antenatal and child welfare clinics. In order to identify knowledge gaps, particularly with regard to EBF, mothers must be thoroughly evaluated during antenatal visits and at child welfare clinics. This will allow for the delivery of more thorough education. Male participation in the care of their infants should be encouraged, and these educational sessions should be expanded to include spouses.

### Strength and limitation

The strength of this study included the determination of maternal knowledge and attitude toward EBF and the incorporation of support from immediate family as well as knowledge about the benefits associated with it. The study was structured based on the theoretical framework of planned behavior by Ajzen [20] and the incorporation of the WHO criteria or guidelines on EBF added strength to the study. The main limitation of this study was the time frame used for data collection and small the sample size which did not allow the inclusion of other relevant variables. This may result in measurement error due to recall errors or social desirability bias. A longitudinal study is therefore recommended to follow up with mothers and babies until they are toddlers to assess the complete benefits of EBF.

## Data Availability

The datasets generated and/or analyzed during the current study are not publicly available due to patient confidentiality but are available from the corresponding author on reasonable request.
